# CD38/cADPR Signaling Pathway in Airway Disease: Regulatory Mechanisms

**DOI:** 10.1155/2018/8942042

**Published:** 2018-02-07

**Authors:** Deepak A. Deshpande, Alonso G. P. Guedes, Richard Graeff, Soner Dogan, Subbaya Subramanian, Timothy F. Walseth, Mathur S. Kannan

**Affiliations:** ^1^Department of Medicine, Thomas Jefferson University Medical School, Philadelphia, PA, USA; ^2^Department of Veterinary Clinical Science, College of Veterinary Medicine, University of Minnesota, St. Paul, MN, USA; ^3^Department of Pharmacology, University of Minnesota Medical School, Minneapolis, MN, USA; ^4^Department of Medical Biology, Yeditepe University School of Medicine, Istanbul, Turkey; ^5^Department of Surgery, University of Minnesota Medical School, Minneapolis, MN, USA; ^6^Department of Veterinary and Biomedical Sciences, College of Veterinary Medicine, University of Minnesota, St. Paul, MN, USA

## Abstract

Asthma is an inflammatory disease in which proinflammatory cytokines have a role in inducing abnormalities of airway smooth muscle function and in the development of airway hyperresponsiveness. Inflammatory cytokines alter calcium (Ca^2+^) signaling and contractility of airway smooth muscle, which results in nonspecific airway hyperresponsiveness to agonists. In this context, Ca^2+^ regulatory mechanisms in airway smooth muscle and changes in these regulatory mechanisms encompass a major component of airway hyperresponsiveness. Although dynamic Ca^2+^ regulation is complex, phospholipase C/inositol tris-phosphate (PLC/IP3) and CD38-cyclic ADP-ribose (CD38/cADPR) are two major pathways mediating agonist-induced Ca^2+^ regulation in airway smooth muscle. Altered CD38 expression or enhanced cyclic ADP-ribosyl cyclase activity associated with CD38 contributes to human pathologies such as asthma, neoplasia, and neuroimmune diseases. This review is focused on investigations on the role of CD38-cyclic ADP-ribose signaling in airway smooth muscle in the context of transcriptional and posttranscriptional regulation of CD38 expression. The specific roles of transcription factors NF-kB and AP-1 in the transcriptional regulation of CD38 expression and of miRNAs miR-140-3p and miR-708 in the posttranscriptional regulation and the underlying mechanisms of such regulation are discussed.

## 1. Introduction

Asthma is an inflammatory disease in which proinflammatory cytokines have a role in inducing abnormalities of airway smooth muscle (ASM) function and in the development of airway hyperresponsiveness (AHR). Airway smooth muscle obtained from asthmatics is different from smooth muscle obtained from healthy subjects [1]. Inflammatory cytokines alter calcium (Ca^2+^) signaling and contractility of ASM, which results in nonspecific AHR to agonists [2–4]. The increased ASM mass and heightened contractile response of ASM in asthmatic subjects contribute to airway narrowing during an asthma episode [5]. The molecular pathogenesis of asthma includes modulation of expression, activity, or sensitivity of intracellular signaling molecules and effector targets in ASM cells. Studies of gene expression in airway biopsies from mild allergic asthmatics reveal increased expression of contractile proteins and proteins involved in the regulation of these proteins, leading to faster velocity of actin filament propulsion and AHR [6]. Furthermore, ASM cells from asthmatics proliferate at a higher rate and are resistant to antimitogenic effect of glucocorticoids, secrete chemokines, and attain a hypercontractile phenotype [7–9]. In this context, Ca^2+^ regulatory mechanisms in ASM and changes in these regulatory mechanisms encompass a major component of AHR. Although dynamic Ca^2+^ regulation is complex, phospholipase C/inositol tris-phosphate (PLC/IP3) and CD38-cyclic ADP-ribose (CD38/cADPR) are two major pathways mediating agonist-induced Ca^2+^ regulation in ASM. We will focus our discussion on CD38/cADPR pathway in this review.

The *CD38* gene encodes a glycosylated ~45 kDa type II transmembrane protein whose enzymatic activity generates cADPR and adenosine diphosphoribose (ADPR) from NAD^+^ and nicotinic acid adenine dinucleotide phosphate (NAADP) from NADP^+^. The cADPR and NAADP regulate Ca^2+^ signaling and contractility in smooth muscle cells [2]. Extracellular ADP-ribosyl cyclase activity of CD38 leads to generation of cADPR outside the cells that is believed to enter cells via membrane channels formed by CD38 dimers or connexin-43 [10, 11]. Interestingly, recent studies have provided evidence for expression of type III CD38 in human multiple myeloma cells wherein the catalytic domain of the molecule is cytoplasmic and amenable to cytosolic regulation [12]. Furthermore, site-directed mutagenesis of the cationic amino acid residues in the amino-terminal region of CD38 results in the conversion from a mixture of type II and type III orientations to predominantly type III. Recombinant expression of the type III CD38 in a heterologous cell system led to elevation of intracellular concentrations of cADPR [13]. Whether type III CD38 is expressed on ASM membranes and its contribution to cADPR-mediated calcium release needs additional investigation.

Using pharmacological and genetic approaches in human cells and tissues, and murine models of asthma, we have demonstrated the contribution of CD38-cADPR to ASM Ca^2+^ signaling and contractility. Airway myocytes from CD38 knockout (CD38KO) mice exhibit attenuated intracellular Ca^2+^ ([Ca^2+^]_i_) responses to agonists [2] and methacholine-induced airway responsiveness in the CD38KO mice is lower than in wild-type (WT) mice. Alteration in CD38 expression and/or modulation of enzyme activities associated with CD38 result in altered Ca^2+^ signaling and contractility of ASM. In murine models of asthma, we demonstrated the role of CD38 in allergen-induced airway inflammation and AHR. CD38KO mice develop attenuated AHR following allergen, IL-13, or TNF-*α* challenge [14–16]. It is important to note that CD38 is also expressed on immune cells that are important in humoral and cell-mediated immune response in asthma [17]. Reconstitution of WT bone marrow in the CD38KO mice partially restores the WT airway phenotype following allergen sensitization and challenge [18]. This suggests that CD38^+^ inflammatory cells that are recruited into the lungs following allergen challenge are sufficient to impart the WT airway phenotype in the CD38KO mice. These findings implicate CD38 in the pathophysiology of asthma. CD38 has been implicated in other human pathologies including in neuroinflammatory diseases, renal dysfunctions, neoplastic disorders, and viral infections (respiratory syncytial virus) [19–22].

**Figure 1:**
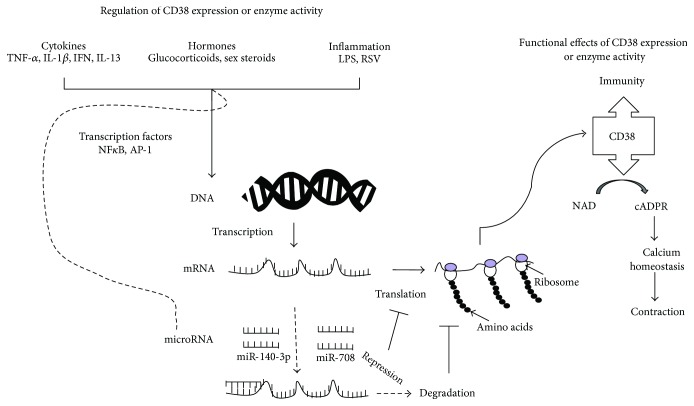
Regulation of expression and functional role of CD38. Bifunctional enzyme CD38 is expressed on a variety of immune and mesenchymal cells including on airway smooth muscle cells. In immune cells, CD38 serves as a cell surface marker and contributes to inflammatory response. CD38 via production of cADPR, a calcium elevating second messenger, contributes to smooth muscle contractility and airway hyperresponsiveness (right half). Regulating CD38 expression, its enzyme activity or cADPR levels in effector cells may lead to human pathologies. Inflammatory cytokines, hormones, and other inflammatory mediators regulate CD38 expression via activation of transcription factors such as NF-*κ*B and AP-1 or via expression of specific microRNAs (left half). Overall, CD38 expression can be modulated at transcriptional level as well as posttranscriptionally by microRNAs.

Inflammatory cytokines such as IL-13 and TNF-*α*, which are implicated in asthma, augment CD38 expression and cADPR-mediated Ca^2+^ release and increase contractility of ASM. ASM cells obtained from subjects who died from an episode of asthma or subjects with a history of stable asthma show significantly enhanced expression of CD38 (both transcript and protein) to low concentrations of TNF-*α* compared to expression in cells from nonasthmatics [5]. This is significant since the TNF-*α* axis does have a role in asthma that is refractory to current therapy, and TNF-*α* levels in bronchoalveolar lavage (BAL) fluid are elevated in patients with severe asthma [23–26]. These observations collectively indicate that the capacity for CD38/cADPR signaling in [Ca^2+^]_i_ regulation and contractility of ASM is significantly greater in asthmatic ASM cells than in cells derived from nonasthmatic subjects. This might arise due to altered CD38 expression and/or modulation of enzyme activities associated with CD38. Therefore, we have examined the transcriptional and posttranscriptional regulation of expression of CD38 in human ASM cells obtained from asthmatics and healthy subjects. Specifically, the role of several transcription factors and miRNAs in the regulation of CD38 expression was examined in these studies. Of note, the contribution of CD38 to [Ca^2+^]_I_ regulation and the underlying mechanisms of such regulation have been addressed in several prior publications and will not be considered here. This review will focus on the role of various transcription factors and specific miRNAs in the regulation of CD38 expression in ASM (Figure 1).

## 2. CD38 Gene Organization, Polymorphisms, and Human Diseases

In the human and mouse, the gene encoding CD38 (*CD38*, human; *cd38*, mouse) is localized on chromosomes 4 and 5, respectively [27]. The CD38 polypeptide is encoded by a >80 kbp length gene comprising 8 exons, more than 98% being represented by intronic sequences. We have cloned the promoter region of *CD38* (Acc. number DQ091293), and sequence analysis reveals putative NF-*κ*B, AP-1, C/EBP*β*, glucocorticoid response element (GRE), and an estrogen response motif [28]. Inducible elements are reported in the 5′ end of intron 1. In addition, intron 1 is reported to have a DR5 repeat, the retinoid acid response element, and a ~900 bp CpG island associated with exon 1 and the 5′ end of intron 1 [29]. Binding of Sp1 to the CpG motif appears to maintain it in an unmethylated state and this allows for constitutive expression of CD38 in cells. These response elements and transactivating regulatory sequences within the *CD38* gene allow for transcriptional regulation by a diverse array of factors and mechanisms [29–31]. In different cell types, CD38 expression is induced by retinoic acid through the retinoic acid response element located within intron 1 of the *CD38* gene [32]. Single nucleotide polymorphisms in regulatory or protein coding regions of *CD38* presumably alter CD38 expression. A polymorphism in intron 1 of the human *CD38* has been described [27]. The absence or presence of a *Pvu*II site defines two alleles, a *CD38*^∗^A allele and a *CD38*^∗^B allele. Ferrero et al. have identified that in the Italian Caucasian population, the *CD38*^∗^B allele appears to be more frequent than the *CD38*^∗^A allele [27]. Okamoto's group has also described a mutation in exon 4 of *CD38* in some subjects with type II diabetes mellitus [33]. CD38 is implicated in the oxytocin signaling pathway, and single nucleotide polymorphisms in this gene have been associated with low serum oxytocin levels in autism spectrum disorder (ASD) patients [19, 34, 35]. Disruption of oxytocin signaling has been associated with features of ASD, including impaired communication and social behavior, based on animal studies. Whether polymorphisms in the *CD38* gene are associated with asthma in humans remain to be determined. In this context, a recent report has identified an individual with autism and asthma with an inherited maternal deletion of 4p15.32 resulting in a BST1-CD38 fusion transcript [36]. This is the first report that describes rearrangements involving CD38 or deletions in patients with ASD.

## 3. Regulation of CD38 Expression in ASM Cells: Role of Transcriptional Mechanisms and Signaling Crosstalk

In prior studies, we demonstrated augmented CD38 expression, ADP-ribosyl cyclase activity, cADPR production, and [Ca^2+^]_i_ responses to agonists in human ASM cells by IL-13 and TNF-*α* [2–4, 15, 16]. Effects of inflammatory cytokines on CD38 expression and cADPR-mediated calcium release in human ASM cells have been studied by other investigators [37–41]. These data suggest that CD38 expression is regulated by cytokines in ASM cells (Figure 1). Numerous studies have unequivocally demonstrated the central role of inflammatory and Th2 cytokines in asthma [42]. Furthermore, mice challenged intranasally with IL-13 or TNF-*α* exhibited significantly greater airway inflammation, BAL cytokine levels, and augmented methacholine-induced changes in airway resistance. These changes in the airways were significantly attenuated in the CD38KO mice compared to responses in the WT mice. These observations strongly implicate CD38 in the pathophysiology of asthma in the mouse models and that IL-13 and TNF-*α* alter ASM functions [43–45] in part via CD38.

Evidence for TNF-*α* in the pathogenesis of allergic airway disease comes from the following observations. Human clinical trials with anti-TNF-*α*-immunomodulators (etanercept) and anti-TNF-*α* monoclonal antibodies (infliximab) have shown that TNF-α has a role in asthma that is refractory to corticosteroids [42]. TNF-α secretion following allergen challenge is higher in atopic asthmatics (i.e., asthmatics with allergy) than in nonatopics (asthmatics with no clearly defined allergy) [46, 47]. Aerosolized TNF-*α* to human volunteers causes AHR [48–50]. In vitro tissue studies have shown that TNF-*α* treatment increases contractility and Ca^2+^ signaling in ASM cells [15]. The effects of TNF-*α* in corticosteroid-resistant asthma may be mediated by direct effects to alter the dynamics of the Ca^2+^ response to agonists and thereby the contractility of the ASM. TNF-*α* induces ASM cell synthesis and release of IL-8 and eotaxin, and upregulates the expression of adhesion molecules, receptors for inflammatory mediators, and growth factors [51, 52]. A polymorphism in the 5′ untranslated region of the TNF gene (G-308A), referred to as TNF-308 GG genotype, is associated with inflammatory diseases including asthma [53].

We have also observed that among the cytokines that are implicated in asthma, TNF-*α* causes the greatest induction of CD38 expression in ASM cells [3, 5]. Furthermore, a single intranasal challenge with TNF-*α* causes significant augmentation of methacholine-induced airway resistance in the WT but not in the CD38KO mice [15]. Therefore, we examined the regulation of CD38 expression in airway myocytes obtained from asthmatic and healthy human subjects in an attempt to identify signaling intermediates involved in this process and to identify potential therapeutic targets. Other investigators have demonstrated that CD38 expression is altered by other cytokines such as type I interferons and lipopolysaccharide (LPS) in many other cell types including epithelial cells, renal cells, macrophages, and other immune cells [19, 22].

Sequence analysis of a 3 kb putative *CD38* promoter fragment (Gen-Bank accession number DQ091293) cloned from a human erythropoietic cell line (K562 cells) in our laboratory revealed binding sites for NF-*κ*B, AP-1, and glucocorticoid receptor (GR). To determine whether CD38 expression in human ASM cells is regulated by TNF-*α* and GRE, we measured the binding of transcription factors and the GR to their respective putative sites within this promoter region [28, 54]. Our results demonstrate that TNF-*α* causes increased binding to the NF-*κ*B site and to 3 of the 6 putative AP-1 sites. Site-directed mutagenesis of the AP-1 site that exhibited very strong binding of nuclear proteins (AP-1 site 4) or the NF-κB site abolished promoter activation induced by TNF-*α*. Regulation of the CD38 gene has also been investigated in human myeloid cells [32]. In these cells, CD38 expression is induced by retinoic acid through the retinoic acid response element located within the first intron of the *CD38* gene. Response elements for other transcription factors, including AP-1, have been described in osteoblasts and osteoclasts [31], and in these cell lines, TNF-*α*-induced activation of a *CD38* promoter fragment requires an intact AP-1 site.

Glucocorticoids are widely used in the management of inflammatory airway diseases [55, 56]. The mechanisms of action of glucocorticoids are complex and involve transcription factors, including NF-*κ*B [56, 57]. The glucocorticoid dexamethasone increased the binding of GR to 3 of the 4 putative GRE sites within the *CD38* promoter and abolished promoter activation induced by TNF-*α* [28, 54]. These results demonstrate that TNF-*α* regulates CD38 expression transcriptionally through NF-*κ*B and AP-1 (Figure 1), and glucocorticoids decrease this expression possibly by binding to GREs within the *CD38* promoter and/or also by decreased NF-κB- and AP-1-mediated transcription [28, 54]. Interestingly, Tliba et al. demonstrated that CD38 augmenting effect of TNF-α in human ASM cells is insensitive to glucocorticoid treatment, and this steroid resistance involves action of interferon regulatory factor (IGF)-1 and glucocorticoid receptor *β* (GR-*β*) isoform [38, 58].

The mechanisms by which mitogen-activated protein (MAP) kinases mediate changes in gene expression are complex and involve the interaction of several proteins and transcription factors. In ASM cells, MAPKs mediate changes in the profile of gene expression through phosphorylation of intracellular proteins including transcription factors, and TNF-*α* causes activation of the MAPKs, that is, p38, JNK, and ERK1/2, resulting in the regulated expression of a variety of genes involved in excitation-contraction coupling [59–65]. NF-*κ*B mediates the effects of TNF-*α* in ASM and other cell types. Repression of transcription of proinflammatory genes by glucocorticoids involves binding to GREs within the promoter region and through interaction with transcription factors. With respect to interaction of GR and regulation through AP-1 and NF-*κ*B, evidence supports direct interaction between the proteins rather than inhibition of binding of transcription factors to their respective response elements [66–71]. GR binding to *cis*-acting elements and the *trans*-acting factors that bind these are essential for a complete glucocorticoid response. The anti-inflammatory effects of glucocorticoids may be distinct from the effects on gene transcription and may involve the MAPK pathways. Glucocorticoids can induce the expression of a dual specificity phosphatase 1 (DUSP1), also known as MAPK phosphatase 1 (MKP-1) [60, 61, 72–74]. MKP-1 causes dephosphorylation and inactivation of the activated MAPKs. Thus, MKP-1 induction is considered as an important anti-inflammatory action of glucocorticoids. MAPKs also regulate the stability of mRNAs for several proinflammatory cytokines. We have systematically examined the role of MAP kinases and glucocorticoids in TNF-*α*-mediated CD38 expression in human cells. We also examined whether the differential induction of CD38 expression in asthmatic ASM cells can be attributed to differential activation of MAP kinases. Exposure to TNF-*α* caused a rapid phosphorylation of all three MAP kinases. Dexamethasone decreased TNF-*α*-induced phosphorylation of the MAP kinases and increased MKP-1 expression. These effects of dexamethasone resulted in significant attenuation of CD38 expression. Furthermore, in cells transfected with MKP-1-specific small interfering RNAs, there was significant attenuation of MKP-1 expression and partial reversal of dexamethasone inhibition of CD38 expression. These findings indicate that regulation of CD38 expression in human ASM cells by glucocorticoids involves decreased signaling through MAP kinases and activation of transcription factors. Glucocorticoid effects on inhibition of CD38 expression involve both transcriptional and posttranscriptional mechanisms, the latter through alteration of transcript stability. In human ASM cells, p38 and ERK MAPKs are involved in CD38 transcript stability, while the JNK MAPK is involved in transcriptional regulation of CD38 expression. The 3′UTR of CD38 mRNA contains A/U rich elements and their role in CD38 mRNA stability remains to be determined.

Phosphatidylinositol-3 kinase/Akt pathway (PI3 kinase) is a key component of intracellular signaling pathways activated by cytokines, growth factors, and glucocorticoids in ASM cell [75, 76]. Binding of TNF-*α* to TNFR1 resulting in the activation of NF-*κ*B and the MAPK signaling pathways results in induction of *CD38* gene [63, 77]. We have examined the role of PI3 kinases in the regulation of CD38 expression and function in human ASM cells [78]. Since our previous studies showed a differential induction of CD38 expression by TNF-*α* in ASM cells derived from asthmatic subjects, we determined whether PI3Ks are involved in this differential induction. Cells were treated with pan-PI3K inhibitors or class I-selective or isoform-selective PI3K inhibitors with and without TNF-*α*. In other studies, we transfected the cells with a catalytically active form of PI3K or PTEN or nontargeting or p110 isoform-targeting siRNAs before exposure to TNF-*α* and measured CD38 expression, ADP-ribosyl cyclase activity, and activation of Akt, NF-*κ*B, and AP-1. TNF-*α*-induced CD38 expression and enzyme activity and Akt activation were inhibited by a pan-PI3K inhibitor. P110 expression by transient transfection increased Akt activation, and basal and TNF-*α*-induced CD38 expression, while PTEN expression attenuated Akt activation and CD38 expression. Silencing of p110α or –*δ* isoform by siRNAs caused a reduction in TNF-*α*-induced CD38 expression in asthmatic and nonasthmatic ASM cells to a comparable magnitude. Furthermore, the PI3K inhibitors had no significant effect on NF-*κ*B or AP-1 activation. These results indicated that in human ASM cells, regulation of CD38 expression is mediated through class I PI3K isoforms, but PI3K signaling may not be involved in the differential induction of CD38 in asthmatic ASM cells.

In addition to the mechanisms of regulation of CD38 in ASM described above, there is also evidence for hormonal regulation in other smooth muscles. In this context, we previously reported estrogen regulation of CD38 expression in myometrium smooth muscle [79–81]. In ovariectomized rats treated with estrogen, there was enhanced expression of CD38 in the myometrium compared to expression in control rats. Furthermore, when we compared CD38 expression in the myometrium obtained from preterm versus term rats, there was significantly greater expression in the term rat myometrium compared to expression in preterm rat myometrium. CD38 expression in preterm myometrium was comparable to expression in myometrium obtained from ovariectomized rats or from ovariectomized rats treated with both estrogen and progesterone. The augmented CD38 expression in the myometrium of term rats or ovariectomized rats treated with estrogen was associated with enhanced ADP-ribosyl cyclase but not cADPR hydrolase activity, suggesting a differential regulation involving posttranslational modification of the protein. However, the nature of this posttranslational regulation of CD38 enzyme activities is not currently known. Other hormones that might be involved in the regulation of ADP-ribosyl activity include betamethasone and L-thyroxine. Treatment of renal cells with thyroxine augments ADP-ribosyl cyclase and cADPR generation and contributes to nephrotoxicity [82]. The role of retinoic acid in modulating CD38 expression and cADPR levels in many cell types has been investigated extensively [32, 83]. Betamethasone, another hormone, administered antenatally in sheep model leads to increased CD38 expression and contributes to postnatal hypertension suggesting a potential augmenting effect of CD38 in peripheral vascular resistance [84].

## 4. Posttranscriptional Regulation of CD38 Expression: Role of MicroRNAs (miRNAs)

miRNAs are noncoding ~22 nt RNAs that have a central role in posttranscriptional regulation of expression of genes involved in inflammation and other cellular functions [85–89]. miRNAs regulate gene expression by destabilizing the transcripts or by translational repression [90]. Evidence has emerged for their role in airway diseases such as asthma, COPD, and idiopathic pulmonary fibrosis [91, 92]. miR-21 appears to have a role in Th1/Th2 immune responses to antigen, suggesting a role in the pathogenesis of allergic asthma. Other investigations have shown that *let-7* and miR-155 regulate IL-13 signaling and miR-133 in RhoA expression, thus highlighting their potential role in airway inflammation and AHR [85–88]. Web-based target prediction algorithms were used to determine potential miRNA response elements in the CD38 3′-untranslated region (3′UTR). Although the target prediction resulted in the identification of several potential miRNA targets in the 3′UTR, quantitative RT-PCR showed expression of miR-140-3p and miR-708. Therefore, we chose to examine the contribution of these two miRNAs to posttranslational regulation of CD38 expression in human ASM cells. We employed luciferase reporter assays to determine miR-140-3p and miR-708 binding to the *CD38* 3′UTR. In human ASM cells transfected with miR-140-3p or miR-708 mimics, CD38 mRNA expression and ADP-ribosyl cyclase activity were significantly attenuated compared to expression and enzyme activity in cells transfected with a control oligonucleotide. Site-directed mutagenesis of the miRNA binding sites on the CD38 3′UTR reversed the inhibition of luciferase activity induced by the miRNA mimics. We then determined whether the differential induction of CD38 in cells from asthmatic airways could be attributed to differential induction of expression of miRNAs. Exposure to TNF-*α* caused a reduction in the expression of miR-140-3p and miR-708 to a comparable magnitude in cells from asthmatics and nonasthmatics. Furthermore, stability of the CD38 transcript was unaltered by overexpression of the miRNAs. These findings suggest that the reduction of CD38 expression may arise to a large extent from indirect mechanisms and that the differential induction of CD38 expression in asthmatic ASM cells may not be attributed to differences in miRNA levels following exposure to TNF-*α* [93, 94] (Figure 1).

Previous studies showed that MAP kinases are involved in the regulation of CD38 expression in human ASM cells. Therefore, we examined the activation of the MAPKs following transfection of cells with the miRNAs. In cells transfected with the miR-140-3p mimic, there was decreased TNF-*α*-induced activation of p38 MAPK and NF-*κ*B. On the other hand, miR-708 transfection caused a reduction in JNK MAPK activation and Akt activation. Transfection with miR-708 also resulted in induction of MKP-1 and *PTEN*, phosphatases that are involved in dephosphorylation of MAPKs and PI3K signaling, respectively [94]. Prior reports have provided evidence that *PTEN* expression in certain cancer cells is regulated by miRNAs [95, 96]. For example, miR-221 and miR-222 inhibit *PTEN* expression by binding to the 3′UTR, while knockdown of these miRNAs increases *PTEN* expression. Furthermore, regulation of *PTEN* expression in these cancer cells has a direct effect on tumor cell proliferation, migration, and invasion. There is evidence that the PI3K/Akt signaling pathway has a major contribution to ASM proliferation in asthmatics [75, 76]. Therefore, we hypothesize that by regulating the expression of PTEN and thereby the PI3K/Akt signaling pathway, miR-708 may have a profound impact on cell proliferation and airway wall remodeling. These findings strongly indicate that indirect mechanisms are involved in the actions of miRNAs in the regulation of CD38 expression and in the control of airway inflammation and ASM proliferation.

## 5. Conclusions

This review outlines the transcriptional and posttranscriptional mechanisms of regulation of CD38 expression and function in ASM. Using the promoter-luciferase transfection system and site-directed mutagenesis approaches, we have demonstrated the critical role of NF-*κ*B and AP-1 transcription factors in such regulation. Cytokines and steroid (glucocorticoids, sex steroids) hormones modulate CD38 expression in ASM. Our studies on CD38 expression in the myometrium show estrogen regulation and inhibition of expression by progesterone. Furthermore, during gestation, CD38 expression in the myometrium is maintained at a relatively low level until very close to term when the ratio of estrogen to progesterone is high. An interesting observation of these studies is that estrogen-mediated augmented CD38 expression causes a differential regulation of the catalytic activities of the enzyme, the mechanisms underlying this being not well defined. In human ASM cells obtained from fatal asthmatics, TNF-*α* induces a significantly greater induction of CD38 expression compared to expression in cells obtained from nonasthmatic subjects. This differential induction of CD38 expression suggests that the capacity for cADPR-mediated calcium regulation is significantly greater in asthmatic airway smooth muscle cells compared to cells from healthy subjects. In this context, our previous studies showed that exposure to TNF-*α* results in significantly greater airway responsiveness to inhaled methacholine in the WT than in the CD38KO mice. Furthermore, CD38KO mice fail to develop an asthmatic airway phenotype following sensitization and challenge with allergen as well as following intranasal challenge with IL-13, a Th2 cytokine implicated in the pathogenesis of asthma. Our recent studies showed that transfer of bone marrow from WT mice to CD38KO mice partially restores the WT phenotype following allergen sensitization and challenge. These findings support the concept that CD38 expressions in airway structural cells and inflammatory cells recruited into the lungs following allergen challenge are both critical for the development of an asthmatic phenotype. The investigations on the posttranscriptional regulation of CD38 expression provide evidence for two specific miRNAs in such regulation. The mechanisms by which miR-140-3p and miR-708 mediate the inhibitory effect on CD38 expression are different. While miR-140-3p effects on downregulation of CD38 expression in human ASM cells are mediated via inhibition of p38 MAPK and NF-*κ*B activation, the effects of miR-708 effects are via inhibition of JNK MAPK and PI3K activation, the latter by increased expression of PTEN. Dysregulation of expression of these two miRNAs should have profound effects on CD38 expression and airway function.
